# CRT-Estimands Framework: consensus based extension of the ICH E9(R1) addendum for cluster randomised trials

**DOI:** 10.1136/bmj-2025-089050

**Published:** 2026-05-21

**Authors:** Brennan C Kahan, Frank Bretz, Andrew Copas, Michael O Harhay, Gary S Collins, Melanie Bahti, Dongquan Bi, Marion K Campbell, Katherine R Courtright, Timothy Feeney, Andrew B Forbes, Amit X Garg, Scott D Halpern, Patrick J Heagerty, Karla Hemming, Sally Hopewell, James P Hughes, David M Murray, Joseph S Ross, Derek Stewart, Monica Taljaard, Obioha C Ukoumunne, Catherine L Auriemma, Fan Li

**Affiliations:** 1 MRC Clinical Trials Unit at University College London, London, UK; 2 Novartis Pharma AG, Basel, Switzerland; 3 Institute of Medical Statistics, Centre for Medical Data Science, Medical University of Vienna, Vienna, Austria; 4 Center for Clinical Trials Innovation, Department of Biostatistics, Epidemiology and Informatics, University of Pennsylvania Perelman School of Medicine, Philadelphia, PA, USA; 5 Department of Applied Health Sciences, School of Health Sciences, College and Medicine and Health, University of Birmingham, Birmingham, UK; 6 Palliative and Advanced Illness Research Center, Department of Medicine, University of Pennsylvania Perelman School of Medicine, Philadelphia, PA, USA; 7 Aberdeen Centre for Evaluation, University of Aberdeen, Aberdeen, UK; 8 Gillings School of Global Public Health, UNC Chapel Hill, Chapel Hill, NC, USA; 9 *The BMJ*, London, UK; 10 School of Public Health and Preventive Medicine, Monash University; 11 Departments of Medicine, Epidemiology and Biostatistics, Schulich School of Medicine and Dentistry, Western University, London, ON, Canada; 12 Department of Biostatistics, University of Washington, Seattle, WA, USA; 13 Applied Health Research, School of Health Sciences, College of Medicine and Health, University of Birmingham, Birmingham, UK; 14 Oxford Clinical Trials Research Unit, Nuffield Department of Orthopaedics, Rheumatology and Musculoskeletal Sciences, University of Oxford, Oxford, UK; 15 National Institutes of Health, Bethesda, MD, USA; 16 Section of General Medicine, Department of Medicine, Yale School of Medicine, New Haven, CT, USA; 17 Department of Health Policy and Management, Yale School of Public Health, New Haven, CT, USA; 18 Patient author, UCL MRC Clinical Trials Unit-Methodological Patient Advisory Group, London, UK; 19 Methodological and Implementation Research, Ottawa Hospital Research Institute, Ottawa, ON, Canada; 20 School of Epidemiology and Public Health, University of Ottawa, Ottawa, ON, Canada; 21 NIHR Applied Research Collaboration South West Peninsula, University of Exeter, Exeter, UK; 22 Department of Biostatistics, Yale School of Public Health, New Haven, CT, USA

## Abstract

In order to assess the relevance of trial results or the appropriate trials methods, interest holders (eg, clinicians, patients, and policy makers) need a clear description of the trial’s research question. To improve clarity and consistency in clinical trials, the International Conference on Harmonisation (ICH) released the ICH E9(R1) addendum, which set out a framework for defining estimands—a precise description of the treatment effect to be estimated. While the ICH E9(R1) addendum has been widely adopted, it primarily focused on individually randomised trials. In contrast, cluster randomised trials, where groups of individuals are randomised, present additional challenges for defining estimands. Therefore, the CRT-Estimands Framework was developed as a consensus based extension of the ICH E9(R1) addendum for cluster randomised trials. This framework provides a set of attributes that should be described when defining estimands in cluster randomised trials, with the objective of improving the clarity of the estimands, and consequently, the research questions, in these trials. This article presents the CRT-Estimands Framework with explanations and examples of how it can be implemented. Adopting the framework will improve the clarity of estimands in cluster randomised trials and facilitate interest holders to make informed decisions from these trials.

A clearly defined research question is essential to ensure the appropriate interpretation of a randomised trial. Firstly, clarity around the research question helps trial investigators to design their trial using appropriate methodology. Secondly, it helps interest holders (including patients, clinicians, funders, regulators, health technology assessment agencies, and journal editors) to appraise the trial, by assessing the question’s relevance and by evaluating whether the trial’s methods, including its design and analysis, are appropriate for the chosen question. However, despite its critical importance, historically many trials do not sufficiently describe their research question.^1^ ^2^

To deal with this issue, the International Conference on Harmonisation released an addendum to their E9 guidance document in 2019 (which we refer to here as the ICH E9(R1) addendum), which set out the estimands framework.^3^ An estimand is a precise description of the treatment effect a trial sets out to estimate.^3^ ^4^ ^5^ ^6^ ^7^ ^8^ ^9^ The estimands framework defines how estimands can be used to help ensure trials are addressing relevant questions and using appropriate methods.^3^ ^7^An adapted version of the estimands framework is shown in box 1.

Box 1 **The estimands framework (adapted from ICH E9(R1) addendum released by the International Conference on Harmonisation)**

1. Specify the estimands of interest for each trial outcome
2. Ensure that design considerations (eg, data collection, trial conduct) and the estimator are aligned with the target estimands. For the estimator, specify:
  - The assumptions required for the estimator to be unbiased for the target estimand
  - Any contextual information to justify these assumptions
3. Evaluate robustness of results to deviations from these assumptions:
  - Check plausibility of required assumptions, if possible (eg, through empirical evaluations of trial data, where applicable)
  - Perform sensitivity analyses to evaluate to what extent departures from required assumptions may impact results.

As part of this framework, the ICH E9(R1) addendum provides five attributes that should be described when defining an estimand: population; treatment conditions (ie, the specific versions of treatment to be compared); endpoint/outcome variable; strategies for handling intercurrent events; and population level summary measure (eg, risk or odds ratios).^3^ Its aim was to enable trial investigators to provide a sufficiently clear description of their research question to allow interest holders, in particular regulatory bodies, to make informed decisions based on trial results. Since its publication, the ICH E9(R1) addendum has been adopted by regulatory agencies around the world,^10^ and has been referenced in major trial reporting guidelines.^11^ ^12^ ^13^ ^14^ ^15^ ^16^ ^17^

Importantly, the ICH E9(R1) addendum was primarily developed with individually randomised trials in mind (where individuals are randomised to treatments), because these trials are most commonly used in the regulatory setting. Cluster randomised trials, in which groups of individuals (such as hospitals, schools, or villages) are randomised, are commonly used to evaluate treatments that necessitate implementation at the cluster level, or have a risk of contamination.^18^ ^19^ ^20^ ^21^ ^22^ There is growing recognition that these trials may have additional considerations when defining estimands that are not explicitly mentioned in the ICH E9(R1) addendum.^23^ ^24^ ^25^ ^26^ ^27^ ^28^ ^29^ Examples of such considerations include: which clusters are part of the target population^25^; how individuals and clusters are weighted in the estimand definition (eg, individual average effect *v* cluster average effect)^23^ ^24^ ^27^ ^30^ ^31^ ^32^; and whether estimands are marginal or cluster specific.^22^ ^23^ ^33^ ^34^ Failure to include these additional considerations in the estimand definition may lead to ambiguous research questions, even when using the framework set out in ICH E9(R1).^25^ ^29^

More specific guidance on defining estimands in cluster randomised trials is needed. We therefore developed a consensus based extension of the ICH E9(R1) addendum for cluster randomised trials (the CRT-Estimands Framework), with the objective of improving the clarity of estimands in these trials. This article describes the methods used to develop the framework and provides explanations of the guidance. Box 2 provides a list of key terms.

Box 2 **List of key terms**

**Baseline**

The definition of baseline differs for clusters compared to individuals. For clusters, baseline will typically be the point at which they are randomised. For individuals, baseline will usually be the point at which they are enrolled (or the point at which they become eligible for the trial, if formal enrolment is not done).

**Collapsible/non-collapsible summary measure**

A summary measure is collapsible if the marginal and cluster specific effects are equivalent (eg, difference in means/proportions); it is non-collapsible if the marginal and cluster specific effects can differ (eg, odds ratio). (This description is based on a definition provided Kahan et al.^23^ Other papers have used slightly different definitions.)

**Cluster randomised trial**

A trial where groups of individuals (eg, hospitals, schools, or villages) are randomised between treatments.

**Cluster crossover trial**

Cluster randomised trial design where clusters are assigned to receive interventions in a specific sequence (eg, intervention then control, or control then intervention).

**Estimand**

A precise definition of the treatment effect a trial aims to quantify.

**Estimator**

The statistical method used to compute the estimate of the treatment effect.

**Estimate**

The numerical value computed by the estimator (eg, an odds ratio of 1.25 would be the estimate).

**ICH E9(R1) addendum**

A regulatory document by the International Conference on Harmonisation that has been adopted by all major national regulatory agencies, which sets out a framework for defining estimands.

**Intercurrent event**

Events after baseline that affect either the interpretation or existence of the endpoint.

**Open cohort trial**

Cluster randomised trial in which individuals can join or leave clusters during the trial.

**Parallel group, cluster randomised trial**

A cluster randomised trial design where clusters are assigned to a single treatment condition for the duration of the trial.

**Potential outcome**

What an individual’s outcome would be under a certain treatment assignment (ie, in a two arm trial, individuals will have two potential outcomes: their potential outcome if assigned intervention; and their potential outcome if assigned control).

**Selection (or recruitment or identification) bias**

When individuals enrol (or are enrolled) based on either their knowledge or the recruiter’s knowledge of their cluster’s assigned treatment.

**Sensitivity analysis**

An analysis designed to evaluate to what extent departures from an estimator’s assumptions affect results.

**Stepped wedge trial**

Cluster randomised trial design where clusters are randomly assigned to switch from control to intervention at a specific time point.

**Treatment**

Used to denote the different treatments or interventions being evaluated in the trial. The terms “treatment” and “intervention” are used interchangeably in this manuscript.

**Summary points**

To interpret and evaluate a randomised trial accurately, readers need a clear understanding of the research question being addressed (ie, the estimand)
The CRT-Estimands Framework provides guidance on defining estimands for cluster randomised trials
The CRT-Estimands Framework contains five attributes that should be described when defining estimands for cluster randomised trials, an expanded table of points to consider when defining estimands, and guidance describing how estimands can be used to align statistical analysis methods and sensitivity analyses with key research objectives
Trial investigators should use the CRT-Estimands Framework when defining estimands for cluster randomised trials to ensure trial objectives are transparent

## Scope of this guidance

The CRT-Estimands Framework sets out the attributes that should be described when defining estimands for cluster randomised trials. This guidance applies to any type of cluster randomised trial, including parallel group, crossover, and stepped wedge designs. This guidance aims to ensure that estimands in cluster randomised trials are clearly described and unambiguous; issues such as which estimands are most appropriate, or the best methods of analysis to estimate different estimands, are out of scope, and thus not considered.

## Development of the CRT-Estimands Framework

Development of the CRT-Estimands Framework was overseen by an executive committee (BCK, CLA, DB, FB, GSC, AC, MOH, and FL) that included members with expertise in cluster randomised trials, estimands, causal inference, and guideline development, and included a member involved in the development of the ICH E9(R1) addendum.

We developed this framework following the EQUATOR Network guidance for developing health research reporting guidelines.^35^ Detailed methods, including a protocol that was published on the Open Science Framework (https://osf.io/a5kdy/), are available in the supplementary material.

Briefly, we started with the five attributes listed in the ICH E9(R1) addendum and set out to identify whether any modifications or additions to these attributes were necessary when defining estimands for cluster randomised trials. We then performed a scoping review of relevant databases to identify a list of items for defining estimands in cluster randomised trials that were not explicitly described as part of one of the five attributes listed in the ICH E9(R1) addendum.^29^ Next, we performed a three round Delphi survey (October 2024 to February 2025, 73 participants) to identify additional items and assess the importance of each item.^36^ Full details of the scoping review and Delphi survey are available elsewhere,^29^ ^36^ and are summarised in the supplementary material. An online expert consensus meeting (7 May 2025; 22 attendees) was then held to establish the final set of items to include in the new guidance. Attendees included statisticians, methodologists, clinicians, trial investigators, journal editors, guideline developers, and a patient representative. Following the consensus meeting, the executive committee developed a draft, which was then circulated to all consensus meeting members for review, and finalised through iterative discussions.

## CRT-Estimands Framework

The CRT-Estimands Framework (table 1) includes all five attributes from the ICH E9(R1) addendum; three attributes were modified, and no new attributes were added. A complete list of differences from ICH E9(R1) is shown in box 3. Brief explanations of each attribute, along with points to consider when defining them, are provided in table 2, with more detailed explanations presented below. Examples of how to apply the framework when defining estimands for cluster randomised trials are provided in table 3. Although we present the attributes in the same order as ICH E9(R1), in practice, the determination of the appropriate attributes is typically an iterative procedure and does not necessarily proceed in a linear manner.

**Table 1:** Attributes of CRT-Estimands Framework for defining estimands in cluster randomised trials

Original ICH E9(R1) attributes	New CRT-Estimands Framework attributes
Population of patients	Population of individuals and clusters
Treatment conditions	Treatment conditions
Endpoint/outcome variable	Endpoint/outcome variable
Strategies to handle intercurrent events	Strategies to handle individual level and cluster level intercurrent events
Population level summary measure	Population level summary measure, including how individuals and clusters are weighted (eg, individual average), and whether summaries are marginal or cluster specific, if applicable

ICH E9(R1)=2019 addendum to the International Conference on Harmonisation’s E9 guidance document on estimands.

Box 3 **Summary of differences in terminology and definitions between the ICH E9(R1) addendum and the CRT-Estimands Framework**

**Extension of attributes**

- Intercurrent events attribute: added clarification that cluster level intercurrent events should be included.
- Population attribute: added clarification that the population of clusters should be described.
- Population level summary measure: added clarification about how individuals and clusters are weighted, and whether summaries are marginal or cluster specific, should be described.

**Changes to definitions**

- The definition for intercurrent events was modified to clarify that events occurring after baseline (rather than treatment initiation) can be considered intercurrent events.

**Changes to terminology**

- The term “individuals” is used throughout instead of “patients” (eg, “population of individuals”).
- The “variable” attribute has been renamed as the “endpoint/outcome variable” attribute.

**Changes to description of the “estimands framework”**

- Some wording or descriptions have been modified.
- Added clarification that assumptions for unbiasedness required by estimator should be explicitly described, as well as any contextual information to justify these assumptions.
- Added clarification that, where possible, empirical evaluations of assumptions should be performed.

ICH E9(R1)=2019 addendum to the International Conference on Harmonisation’s E9 guidance document on estimands.

**Table 2:** Explanation and points to consider when defining estimand attributes for cluster randomised trials

Attribute	Explanation	Points to consider
Population of individuals and clusters	Which individuals and clusters are targeted by the research question	Which individuals and clusters do researchers want to estimate the treatment effect for (eg, students in a year 7 class (age 11-12 years) in schools with at least 200 pupils)
		Are all individuals and clusters of interest, or only a subpopulation (eg, only individuals who would adhere to their assigned treatment, or clusters that would implement the intervention if assigned)
		If some individuals do not enrol in the trial because of their cluster’s assigned treatment (ie, selection or recruitment/identification bias), does interest lie in all eligible individuals, or some subpopulation of individuals who would enrol based on the assigned treatment?
Treatment conditions	Which interventions are of interest for the comparison	Which intervention strategies do researchers want to compare (eg, implementation of a quality improvement programme *v* usual care)
		Is interest in understanding how interventions work regardless of whether they are always delivered correctly, or if they had been implemented and administered as intended?
		If duration of exposure to interventions varies across individuals or clusters, does interest lie in the intervention’s effect at a specific point, or in some average across the various levels of exposure?
Endpoint/outcome variable	Which outcome is to be obtained for each individual to answer the research question	Endpoints may be defined in relation to the occurrence of an intercurrent event such as death (eg, average quality of life up to 12 months or death, whichever is first (while-alive strategy)), or the occurrence of an intercurrent event may be incorporated into the endpoint (eg, hospital admission or death within three months (composite strategy))
Strategies to handle individual level and cluster level intercurrent events	Strategies used to handle each intercurrent event* in the treatment effect definition	Intercurrent events can occur at the individual level (eg, if individuals stop taking their assigned treatment) or the cluster level (eg, if hospitals stop delivering the assigned intervention)
		Precise specification of intercurrent event strategies can help clarify the population of interest (eg, all individuals *v* those who would adhere to their assigned treatment), the versions of treatment being compared (eg, interventions regardless of non-adherence *v* interventions under 100% adherence), and the endpoint (eg, quality of life at 12 months *v* quality of life at 12 months or up to the point of the intercurrent event, whichever is first)
		Cluster randomised trials may have unique intercurrent events, such as when individuals leave or change clusters, or when clusters merge together or split apart during the trial
Population level summary measure, including how individuals and clusters are weighted (eg, individual average), and whether summaries are marginal or cluster specific, if applicable	How endpoints will be summarised and compared between treatments conditions	An overall summary of each individual’s potential outcome is obtained under each treatment condition (eg, the mean), and these summaries are then compared, for example, through a risk or odds ratio or difference in means/proportions
		Outcomes can be weighted differently when obtaining overall summaries, for example, by giving each individual or cluster equal weight (frequently referred to as individual average and cluster average effects). When clusters are given equal weight, individuals in larger clusters contribute less to the summary than individuals in smaller clusters
		Summaries can be obtained in different ways, for example, by taking the mean/proportion across all individuals (marginal summary measure), or by contrasting summaries within each cluster then averaging over these contrasts (cluster specific summary measure)

* Intercurrent events are events after baseline that affect the interpretation or existence of the endpoint.

**Table 3:** Examples of defining estimands in cluster randomised trials

Item	Example 1 (adapted from the TRIGGER^37^ trial)	Example 2 (adapted from the BETR^38^ trial)	Example 3 (adapted from the MENISCUS^39^ trial)
Trial description	Parallel group cluster randomised trial comparing liberal versus restrictive transfusion strategy to prevent further bleeding in patients with acute upper gastrointestinal bleeding. Because patients are enrolled after hospitals are randomised, selection/recruitment bias could occur	Cluster randomised crossover trial comparing standard terminal disinfection (bleach) with enhanced terminal disinfection (bleach and ultraviolet light) to disinfect hospital rooms between patients	Parallel group, cluster randomised trial comparing a school based, multi-component, menstrual health intervention versus usual care on mental health scores
Estimand			
Population of individuals and clusters	Adult patients with a new presentation of acute upper gastrointestinal bleeding admitted to a hospital with 24 hour endoscopy and on-site access to intensive care and surgery. All eligible patients are of interest, regardless of whether they would enrol or not	Patient admitted to a previously contaminated, single-patient room in a tertiary, community, or Veteran Affairs hospital	Year 10 (age 14-15 years) female students attending a school at year end with both male and female students, with enrolment of between 40 and 125 year 10 students
Treatment conditions	Red blood cell transfusion when haemoglobin levels drop below 80 g/L versus transfusion when haemoglobin levels drop below 100 g/L, regardless of adherence to the transfusion protocol	Enhanced terminal disinfection (bleach and ultraviolet light) versus standard terminal disinfection (bleach), if interventions were implemented correctly (ie, ultraviolet light always used for enhanced terminal disinfection, and never used for standard terminal disinfection)	Multi-component menstrual health intervention versus usual care, regardless of adherence to the intervention
Endpoint/outcome variable	Further bleeding up to day 28 or death	*Clostridioides difficile* infection during hospital admission	Mental health score at end of school year, as measured by the Strengths and Difficulties Questionnaire (SDQ-25) for total difficulties (range 0-40)
Strategies to handle individual level and cluster level intercurrent events	Non-adherence (receiving or not receiving transfusion against protocol): treatment policy strategy (ie, the endpoint is used whether non-adherence occurred or not) Mortality: while-alive strategy (ie, the endpoint before death is used)	Ultraviolet light not used in the enhanced terminal disinfection (bleach and ultraviolet light) group: hypothetical strategy (ie, the effect if ultraviolet light had been used) Ultraviolet light used in the standard terminal disinfection (bleach alone) group: hypothetical strategy (ie, the effect if ultraviolet light had not been used)	School level non-adherence (ie, schools not implementing the intervention): treatment policy strategy (ie, the endpoint is used whether non-adherence occurred or not) Student level non-adherence (ie, students not attending the intervention components): treatment policy strategy (ie, the endpoint is used whether non-adherence occurred or not) Students switching, leaving, or joining schools during the school year: principal stratum strategy (ie, the effect for students who would be attending a school at the end of the school year under either treatment assignment, regardless of whether it was their original school) Exposure to the intervention for students starting in a usual care school (eg, due to switching from a usual care to intervention school): treatment policy strategy (ie, the endpoint is used whether usual care students were exposed to the intervention or not)
Population level summary measure, including how individuals and clusters are weighted (eg, individual average), and whether summaries are marginal or cluster specific, if applicable	Marginal, individual average odds ratio	Cluster average difference in proportions*	Individual average difference in means*
Summary description of estimand	The primary estimand is the marginal, individual average odds ratio for further bleeding up to 28 days or death between restrictive versus liberal red blood cell transfusion strategies, regardless of adherence to the transfusion policy, in all eligible adults with a new presentation of acute upper gastrointestinal bleeding admitted to a hospital with 24 hour endoscopy and on-site access to intensive care and surgery	The estimand is the cluster average difference in proportions of *Clostridioides difficile* infections between standard terminal disinfection (bleach) versus enhanced terminal disinfection (bleach and ultraviolet light), if implemented correctly (ie, ultraviolet light always used for enhanced terminal disinfection, but not for standard terminal disinfection), in patients admitted to a previously contaminated single-patient room in a tertiary, community, or Veteran Affairs hospital	The estimand is the individual average difference in means for the SDQ-25 score at the end of the school year between a school based multi-component menstrual health intervention versus usual care, regardless of whether schools or students adhere, in year 10 female students attending a school at year end with both male and female students, with enrolment of between 40 and 125 year 10 students. Students leaving or switching schools is handled using a principal stratums strategy, where any student who would be attending a school at the end of the school year, regardless of whether it was their original school, is of interest

Examples have been adapted based on real trials, although some details have been changed.

* The marginal versus cluster specific distinction is not relevant in this trial because it uses a collapsible summary measure (ie, a difference).

## Explanations of attributes

### Population of individuals and clusters

This attribute describes which individuals and clusters are targeted by the research question (ie, which individuals in which clusters investigators want to estimate the treatment effect for). It may be the same population as defined by the trial’s inclusion/exclusion criteria for individuals and clusters but could also be different. For instance, interest may lie in estimating the treatment effect for a subpopulation of either individuals or clusters. The subpopulation could be defined by an observed subgroup (eg, individuals with a specific biomarker at baseline, or clusters of a certain size), or by a principal stratum subpopulation defined based on the potential occurrence of an intercurrent event after baseline (see box 4 for a more detailed explanation, and the section below on Strategies to handle individual level and cluster level intercurrent events).^3^ ^7^ ^40^ ^41^

Box 4 **Illustrations of different intercurrent event strategies**

The strategies outlined here can be used to deal with either individual level or cluster level intercurrent events, and are the same strategies outlined in the ICH E9(R1) addendum. The different intercurrent event strategies affect how potential outcomes from individuals who experience the event are incorporated into the estimand definition. Potential outcomes denote what an individual’s outcome would be under a certain treatment assignment.

In any randomised trial, individuals will have at least two potential outcomes:

- Their potential outcome if assigned to the intervention.
- Their potential outcome if assigned to the control.

Only one of these potential outcomes can be observed (eg, for individuals assigned the intervention, their potential outcome under control cannot be observed). Causal estimands for randomised trials are based on contrasts of potential outcomes (eg, the difference in the mean potential outcome if all individuals were assigned the intervention *v* if all individuals were assigned the control)—that is, they deal with what happens if the same individuals were assigned intervention versus control.

Under each intercurrent event strategy, individuals who experience the intercurrent event are handled differently. In table 4 below, individuals are classified into four distinct stratums on the basis of whether they would experience the intercurrent event under the intervention or control.

**Table 4:** Subpopulations of individuals based on their experience of potential intercurrent events (IE) under different treatment assignments

Treatment assignment	Individual would experience IE	
	If assigned to intervention	If assigned to control
1) Never-IE	No (A)	No (B)
2) Intervention-IE	Yes (C)	No (D)
3) Control-IE	No (E)	Yes (F)
4) Always-IE	Yes (G)	Yes (H)

In this table, stratums 1-4 denote whether individuals would experience the intercurrent event under one or both treatment conditions, with cells A-H denoting a collection of potential outcomes for all individuals in a specific group under each treatment assignment.

For instance, stratum 1 (never-IE) includes individuals who would not experience the intercurrent event regardless of whether they were assigned to intervention or control; stratum 2 (intervention-IE) includes individuals who would experience the intercurrent event if assigned the intervention, but not if assigned to control. Cell A denotes potential outcomes for all individuals in stratum 1 if they were assigned to the intervention; cell B denotes the potential outcomes for those same individuals in stratum 1 if they were assigned to the control. Below, we briefly explain how these stratums and cells are used differently across the different intercurrent event strategies:

- Treatment policy strategy: The intercurrent event is considered irrelevant, and so individuals’ potential outcomes are used across all stratums 1-4, regardless of whether they experience the intercurrent event or not.
- Composite strategy: The intercurrent event is incorporated into the endpoint definition, and so potential outcomes for individuals in cells C and F-H are modified (eg, set to an unfavourable value of the endpoint) as part of the composite endpoint. Potential outcomes for individuals in other cells are unmodified.
- While-on-treatment strategy: The endpoint before the occurrence of the intercurrent event is of interest, and so potential outcomes for individuals in cells C and F-H are used up to the point the intercurrent event occurs. Potential outcomes for individuals in other cells are unchanged.
- Hypothetical strategy: The endpoint pertaining to a hypothetical setting where the intercurrent event would not occur is of interest, and so potential outcomes for individuals in cells C and F-H are replaced with those individuals’ potential outcomes if they had hypothetically not experienced the intercurrent event.
- Principal stratum strategy: The effect in a subpopulation of individuals defined by the occurrence of the intercurrent event is of interest, and so only individuals in certain stratums are included in the population of interest.
  - For instance, interest may lie in the effect of treatment in those who would not experience the intercurrent event under either treatment assignment (ie, the never-IE stratum). Here, the estimand would be based on a contrast of cell A versus cell B (and individuals in stratums 2-4 are not included in the subpopulation for which the estimand is defined).
  - Alternatively, interest may lie in those individuals who would not experience the intercurrent event if assigned to the intervention, regardless of what they would experience the event if assigned to the control. Here, the estimand would include individuals in stratums 1 and 3, and would be based on a comparison of cells A and E versus B and F.

In summary, the first four strategies use the full population (stratums 1-4), either with the original outcomes (treatment policy), modified outcomes (composite, while-on-treatment), or hypothetical outcomes if the intercurrent event had not occurred (hypothetical). The last strategy (principal group) uses the original outcomes within a subpopulation (eg, stratum 1, stratums 1 and 3).

ICH E9(R1)=2019 addendum to the International Conference on Harmonisation’s E9 guidance document on estimands.

As an example of a principal stratum population, investigators may wish to estimate the treatment effect in the subpopulation of individuals who would adhere to the treatment regardless of which treatment they were assigned to.^42^ Alternatively, interest may also lie in the subpopulation of cluster adherers, where investigators want to estimate the effect for individuals attending a cluster that would correctly implement the intervention.

In some cluster randomised trials, individuals are enrolled after clusters have been randomised. Individuals or those recruiting them may therefore be aware of the cluster's treatment assignment, which can lead to selection bias (also known as recruitment or identification bias), where some individuals may enrol if they would receive the intervention but not the control, or vice versa. In this situation, investigators may use analytical methods to attempt to remove the impacts of such bias. Such analytical methods may estimate the treatment effect in different populations of individuals compared with the treatment effect if the analytical method had not been used (eg, based on the subpopulation of individuals who would enrol regardless of what treatment they would receive).^43^ ^44^ Therefore, for trials that may be affected by such selection or recruitment bias, researchers should clarify which specific population of individuals is of interest. In many trials, this population may include all eligible individuals, regardless of whether they would enrol into the trial or not. However, in some cases, investigators may try to estimate the effect within a subpopulation of individuals based on their potential enrolment status under each treatment assignment (eg, the subpopulation of individuals who would enrol regardless of which intervention they would receive).^45^

### Treatment conditions

This attribute describes which versions of treatments are of interest for the comparison. In addition to the treatments being randomised, this description should include:

- How additional interventions beyond the trial treatment, such as concomitant care or rescue medication, are to be handled in the estimand (eg, whether interest lies in comparing treatment A with rescue medication as indicated to treatment B with rescue medication as indicated; or comparing treatment A without rescue medication to treatment B without rescue medication).
- How intercurrent events such as treatment non-adherence are to be handled (eg, whether interest lies in comparing treatment A regardless of non-adherence to treatment B regardless of non-adherence; or comparing treatment A under perfect adherence to treatment B under perfect adherence). (For a more detailed explanation of intercurrent events, see section below on Strategies to handle individual level and cluster level intercurrent events.)

In some cluster randomised trials, individuals or clusters may be exposed to the intervention for different durations owing to the trial design. While this exposure can occur for any cluster randomised trial design, it most frequently occurs in stepped wedge trials; in parallel group, cluster randomised trials with staggered recruitment of clusters; or in open cohort, cluster randomised trials (see box 2 for definitions). Because the effect of some interventions may depend on the duration of clusters or individual exposure, investigators should clarify how the duration is handled in the estimand (eg, whether interest lies in the effect of the intervention after it had been implemented for a certain period, the average effect across a specific timeframe).^46^ ^47^ ^48^ ^49^ ^50^ This issue, along with methods for handling it in the estimand definition, are more fully described in box 5.

Box 5 **Exposure duration for individuals and clusters**

In some cluster randomised trials, individuals and/or clusters may be exposed to the intervention for different durations, and the intervention’s effect may depend on the duration of exposure. For example:

- Some interventions may take time to have an impact on outcomes, for example, a quality improvement intervention to change a clinician’s behaviour may take time to have an effect. Therefore, individuals who enrol a short time after the intervention was first implemented may experience less benefit than individuals who enrol a long time afterwards
- Individuals may join a cluster midway through a trial. For example, in trials in care homes, some residents may live in the care home at the trial outset, but others may join at any time during the trial. For a sustained intervention, such as weekly group activities to reduce loneliness, residents who joined the care home late will have less exposure to the intervention than longer term residents and thus may experience different benefit.

Some examples of settings where variability in exposure durations across clusters can occur are:

- Stepped wedge trials: in this design, clusters are assigned to switch to the intervention at different points during the trial, so that clusters that switch late will implement the intervention for only a short time during the trial (eg, one month) while clusters that switch early will implement it for a long time (eg, one year).
- Parallel group, cluster randomised trials with staggered recruitment of clusters: under staggered recruitment, where not all clusters open at the same time (but all clusters stay open until the trial end date), some clusters will begin the trial early and therefore take part in the trial for a longer period than trials that open late.

Variability in exposure durations across individuals can occur in:

- Open cohort designs: in these designs of open cohorts, individuals can join the cluster throughout the trial period. Individuals who join early will be exposed to the intervention for longer than individuals who join late.

Exposure duration is not an intercurrent event, and differs from treatment discontinuation (which is an intercurrent event), where some individuals or clusters stop taking (or delivering) the assigned intervention. The difference is that under treatment discontinuation, individuals or clusters have received/delivered the intervention for a shorter duration than intended. However, under varying exposure durations, individuals/clusters do receive/deliver the intervention for the intended duration, it is just the intended duration that will vary across individuals and/or clusters, often because of the trial design.

When exposure durations vary, investigators should define the exposure time(s) of interest as part of the treatment conditions attribute. For instance, investigators may be interested in a comparison:

- Between treatment conditions over a specified timeframe (eg, the average effect if the intervention were implemented across all clusters for one year)
- Between treatment conditions at a specific exposure duration (eg, the effect once the intervention has been implemented for six months)
- Between treatment conditions that average across different exposure durations (eg, the average effect when averaged across the exposure duration for each cluster and/or individual).

### Endpoint/outcome variable

This attribute describes which outcome is to be obtained for each individual or cluster to answer the research question. This description should include the relevant timeframe and relevant measurement scales if applicable (eg, quality of life at three months measured using the EQ-5D questionnaire). It should also clarify if the endpoint is to be defined at the level of the cluster, rather than at the level of the individual (eg, if the endpoint is defined as the total number of antibiotic prescriptions made from each general practitioner office).

Endpoints may also be defined in relation to the occurrence of an intercurrent event, such as treatment discontinuation or death (see section below on Strategies to handle individual level and cluster level intercurrent events).^3^ ^7^ ^51^ The while-on-treatment/while-alive strategy changes the timeframe of interest so that it is up to the point of the intercurrent event (eg, average quality of life up to 12 months or occurrence of death, whichever is first) (box 4). The composite strategy incorporates the intercurrent event into the endpoint definition (eg, hospital admission or death within 3 months) (box 4).

### Strategies to handle individual level and cluster level intercurrent events

In individually randomised trials, intercurrent events are events after randomisation that affect the interpretation or existence of the endpoint.^1^ ^2^ ^3^ ^4^ ^5^ ^6^ ^7^ ^8^ ^9^ ^52^ ^53^ ^54^ ^55^ ^56^ Common examples of intercurrent events include treatment discontinuation, treatment switching, receipt of rescue therapy, or mortality when it precludes the existence of the endpoint (eg, if an individual dies at week 6, their pain score at week 12 does not exist).

In cluster randomised trials, intercurrent events can occur at the level of the individual or the cluster. For instance, individuals may decide to stop taking the assigned treatment (individual level intercurrent event) or the cluster may decide to stop delivering the assigned treatment (cluster level intercurrent event).

A challenge in identifying intercurrent events in cluster randomised trials is that, unlike in individually randomised trials, individuals are not randomised. Thus, while cluster level intercurrent events can be defined as events that occur after the cluster was randomised, this timeframe is generally not helpful for identifying intercurrent events at the individual level. This difference is because there may be a large time lag between when a cluster is randomised and when an individual presents to that cluster for treatment, and events that occur during this period would not be considered as intercurrent events, because this period would typically be defined as the individual’s baseline period for the trial.

Therefore, in cluster randomised trials, the timeframe for defining intercurrent events will be different for individual level and cluster level events. For a cluster, intercurrent events are defined as events occurring after the cluster has been randomised, while for an individual, intercurrent events are defined as events occurring after the individual’s baseline period (ie, at the point when treatment would be assigned for the individual, which would typically coincide with the point at which the individual would be randomised in an individually randomised trial). For most trials, this point is when individuals are enrolled. However, some trials do not formally enrol individuals (eg, trials with a waiver of consent in which electronic health records are accessed for all eligible individuals). In these trials, baseline may be the point that individuals become eligible for the trial.

For simplicity, we define both individual level and cluster level intercurrent events as events after baseline that affect the interpretation or existence of the endpoint. However, the definition of baseline differs for cluster level and individual level events: it is defined as the point of randomisation for cluster level events, and as the point of enrolment (or the point when individuals become eligible) for individual level events. 

Both intercurrent events at the individual and at the cluster level can be handled in the estimand definition using the five strategies outlined in the ICH E9(R1) addendum:

- Treatment policy: The intercurrent event is considered irrelevant, and the endpoint is used whether or not the intercurrent event occurs
- Composite: The intercurrent event is incorporated into the definition of the endpoint
- While on treatment/while alive: The endpoint before the occurrence of the intercurrent event is of interest
- Hypothetical: The endpoint pertaining to a hypothetical scenario where the intercurrent event would not occur is of interest
- Principal stratum: The effect in a subpopulation of individuals or clusters who would (or would not) experience the intercurrent event is of interest.

Illustrations of these strategies are provided in box 4. Although cluster randomised trials are often affected by the same types of intercurrent events seen in individually randomised trials, such as treatment discontinuation, they can be affected by unique intercurrent events that do not typically occur in individually randomised trials. These intercurrent events, and methods for handling them in the estimand definition, are described in box 6.

Box 6 **Unique intercurrent events for cluster randomised trials**

**Cluster level non-adherence**

Some clusters may not implement the intervention as intended, which may affect some or all individuals within the cluster. Examples include clusters that:

- Do not implement the assigned intervention
- Delay implementation of the assigned intervention, such that some enrolled individuals do not receive it or receive it for a shorter duration than planned
- Stop delivery of the assigned intervention early
- Deliver a non-assigned intervention (eg, treatment switching, or delivery of non-trial treatments).

However, sometimes delays or non-implementation of the assigned intervention may not constitute an intercurrent event—a distinguishing factor will usually be whether enrolled individuals are affected. For instance, in a parallel group, cluster randomised trial, a cluster that was scheduled to begin implementing the intervention in January may delay this until June. However, so long as no individuals were enrolled before the new start date for implementation (ie, enrolment is also pushed back to coincide with the intervention delivery), this would not be an intercurrent event.

Conversely, in a stepped wedge trial, a delay in implementing the intervention past the scheduled point would affect some enrolled individuals, because the start date to begin enrolment is decided at the trial outset and cannot typically be delayed, so this would constitute an intercurrent event.

**Individuals who leave or change clusters**

In some cluster randomised trials, some individuals may leave or change clusters during the trial. For instance, a student may drop out of school midway through the school year, or transfer from one school to another.

Leaving a cluster is not always an intercurrent event. For instance, for trials conducted in hospitals it is usually intended that patients are discharged at some point. Therefore, such an event does not affect interpretation of that individual’s outcome, and thus is not an intercurrent event. Conversely, it is usually not intended for students to drop out of school midway through the school year. Therefore, this event would affect interpretation of that student’s outcome, and so would be an intercurrent event.

Any of the standard intercurrent event strategies can be applied. For instance, for students leaving schools, a treatment policy strategy could be used to denote that the interest lies in the students’ outcome regardless; a composite strategy to assign students who leave a bad outcome; a while-on-treatment strategy for students’ outcomes before leaving; hypothetical strategy to denote interest in the students’ outcomes had they not left; or a principal stratum strategy to denote interest in the population of students who would remain in school until the school years end under either treatment (or some other population if appropriate).

Changing between clusters would typically be an intercurrent event, because this event is usually not intended. Investigators should consider several issues when these events occur:

- If individuals are exposed to a different intervention than the one assigned in their new cluster (ie, treatment switching), an intercurrent event strategy should be specified to handle this unintended exposure
- Even if individuals who switch are not exposed to different treatments, switching clusters in itself may affect outcomes, and thus an intercurrent event strategy should be specified for this. The same strategies described above can be used; however, different principal stratum populations could be defined (eg, the subpopulation of individuals who would still be in their original cluster at the trial’s end; the individuals who would still be in a cluster at trial’s end, regardless of whether it is their original cluster)
- If a cluster specific summary measure is to be used, investigators may need to define which cluster individuals belong to for this summary (eg, original or final); however, depending on the intercurrent event strategy used, this distinction may not be relevant (eg, for composite, while-on-treatment, or hypothetical strategies, individuals are inherently defined as belonging to their original clusters, because these strategies do not consider outcomes that occur post-switching).

Typically, individuals joining a cluster part way through a trial (where they had not previously belonged to another cluster) is not an intercurrent event (box 5).

**Clusters that merge together or split apart**

In some trials, clusters may merge together (eg, two schools merging such that all students from both schools begin to attend a single school) or split apart (eg, when a new hospital opens near an existing hospital, and the catchment areas change such that some individuals who used to attend the existing hospital now attend the new hospital).

The point at which clusters split apart can be viewed as the end of the original cluster, with two (or more) clusters emerging from this point forward. There are several issues to consider:

- Are the new clusters part of the population of interest? Are outcomes from individuals in new clusters relevant to the research question? These questions will help guide the choice of intercurrent event strategy.
- If new clusters receive different treatment to the original clusters (eg, a non-assigned treatment), an intercurrent event strategy would need to be defined to handle this switch in treatments (which may be in addition to the strategy specified to handle the split itself).

Any of the standard intercurrent event strategies can be used. For example, a treatment policy strategy could be used to denote that outcomes for individuals in the new clusters are relevant; a composite strategy to assign specific outcome values to individuals in the new clusters; and a while-on-treatment strategy to denote interest is in outcomes for individuals in the original cluster only.

Similarly, the point when two or more clusters merge can be viewed as the end of these original clusters, with a new cluster emerging after this point. The same considerations for when clusters split also apply when they merge, and an intercurrent event strategy should be used to clarify the research question of interest.

### Population level summary measure, including how individuals and clusters are weighted (eg, individual average), and whether summaries are marginal or cluster specific, if applicable

This attribute describes how endpoints are summarised and compared between treatment conditions. Two key considerations are how potential outcomes will be summarised (eg, mean, median, proportion), and how these summaries will be contrasted between treatment conditions (eg, difference, risk ratio, odds ratio).

Cluster randomised trials also have several additional aspects that affect how the summary measure is obtained and are therefore important to describe. These factors include: how individuals and clusters are weighted^23^ ^24^ ^30^ ^31^ ^32^; and whether summaries are marginal or cluster specific (described below).^22^ ^23^ ^33^ ^34^

#### How individuals and clusters are weighted

In cluster randomised trials, individuals and clusters can be weighted differently when defining an overall summary.^23^ ^24^ ^27^ ^30^ ^31^ ^32^ For instance, each individual could be considered equally important and thus given equal weight in the summary. This approach is referred to as the individual average (or participant average) effect, and is the effect typically used in individually randomised trials.^31^ As another example, interest may lie in how the intervention affects the clusters, and so each cluster may be considered equally important and is given equal weight in the summary. This approach is referred to as the cluster average effect; here, individuals in larger clusters contribute less to the summary than individuals in smaller clusters.^31^

A hypothetical example illustrating these two approaches is shown in box 7. Other approaches to weighting individuals and clusters are also possible. For instance, individuals could be weighted according to prespecified time periods within clusters (eg, the cluster period average effect).^57^ ^58^ For example, investigators could specify that each one month period within each cluster is allocated equal weight. While this approach is most commonly used in longitudinal cluster designs, such as stepped wedge or cluster crossover designs, it could also be used in parallel group, cluster randomised trials.

Box 7 **Difference between individual average and cluster average effects: illustrative example**

Table 5 presents a hypothetical population to illustrate the difference between the individual average effect and the cluster average effect.

**Table 5:** Hypothetical population to illustrate the difference between individual average and cluster average effects

Cluster	No of individuals within cluster	Treatment effect in cluster*
1	10	5
2	10	5
3	100	1
4	100	1

* Mean difference in potential outcomes under intervention versus control across all individuals within the cluster.

This population contains four clusters and 220 individuals. There are:

- Two small clusters, with 10 individuals each; and
- Two large clusters, with 100 individuals each.

The treatment effects are different in the small clusters compared to the treatment effect in the large clusters (the difference in means is 5 in the small clusters, and 1 in the large clusters).

- Individual average effect: Each individual is counted the same. Because 20 individuals belong to a cluster with an effect of 5, and 200 individuals belong to a cluster with an effect of 1, the overall individual average effect is: ((20×5)+(200×1)) ÷ 220 = 1.4.
- Cluster average effect: Each cluster is counted equally. Two clusters have an average treatment effect of 5, and two clusters have an average effect of 1. Therefore, the overall cluster average effect is: ((2×5)+(2×1)) ÷ 4 = 3.

In the hypothetical population in table 5, the treatment effect is different in small clusters versus the larger clusters—this is an example of informative cluster size, whereby either outcomes or treatment effects differ according to the number of individuals in each cluster. The presence and magnitude of informative cluster size can affect to what degree different approaches to weighting individuals and clusters can yield different answers.^31^

#### Whether summaries are marginal or cluster specific

For both individual average effects and cluster average effects (or other weighting schemes), different summary measures can be obtained depending on the order in which potential outcomes are summarised. For example, a summary measure such as the individual average odds ratio can be defined as either marginal or cluster specific.

A marginal (sometimes referred to as population average) summary measure involves summarising potential outcomes for each treatment condition across all individuals in the population, irrespective of cluster. These marginal summaries are then contrasted between treatment conditions.^23^ A cluster specific (sometimes referred to as conditional) summary measure involves summarising potential outcomes for each treatment condition within each cluster first and then contrasting these summaries to obtain cluster specific contrasts. An average is then taken across these cluster specific contrasts to obtain an overall treatment effect.^23^ An example illustrating these two approaches is shown in box 8.

Box 8 **Difference between marginal (population average) and cluster specific (conditional) summary measures: illustrative example**

Table 6 presents a hypothetical trial to illustrate the difference between marginal and cluster specific summary measures.

**Table 6:** Hypothetical trial illustrating the difference between marginal and cluster-specific summary measures

Trial group	No (%) of events/cluster size		Within-cluster odds ratio
	If assigned intervention	If assigned control	
Cluster 1	90/100 (90)	50/100 (50)	9.0
Cluster 2	50/100 (50)	10/100 (10)	9.0
Overall	140/200 (70)	60/200 (30)	**—**

This trial contains two clusters each with 100 individuals and shows the potential outcomes under both treatment conditions. Below, we use an odds ratio to illustrate the difference between marginal and cluster specific effects. The odds ratio within each cluster is 9.0, although the event rate in each cluster is different (50% event rate under control in cluster 1, and 10% event rate under control in cluster 2).

- Marginal summary measure (also known as population average): a marginal summary measure compares the treatment conditions at the margins of table 6 (ie, contrasts the overall event rates of 70% *v* 30% across the treatment conditions). Formally, it is obtained by^23^:
  - summarising the potential outcomes for each treatment condition across all individuals in the population (to obtain 70% under the intervention and 30% under the control); and
  - contrasting these marginal summaries between treatments (ie, by calculating the marginal odds ratio as (0.7÷(1−0.7)) ÷ (0.3÷(1−0.3)) = 5.4,^23^ which can be interpreted as indicating that the odds of an event would be 5.4 times higher across the population under assignment to intervention *v* control).
- Cluster specific summary measure (also known as conditional): a cluster specific measure averages across the cluster specific treatment effects within each cluster. Formally, it is obtained by^23^:
  - summarising and contrasting the potential outcomes within each cluster first (to obtain cluster specific odds ratios of 9.0 in clusters 1 and 2); and
  - obtaining an overall treatment effect by averaging across the cluster specific contrasts (because each cluster specific contrast is 9.0, then the overall cluster specific odds ratio is also 9.0,^23^ which can be interpreted as indicating that across the population, the average odds ratio within clusters of assignment to intervention *v* control is 9.0).

The distinction between marginal and cluster specific summary measures is only relevant for non-collapsible summary measures, such as an odds ratio. For collapsible summary measures, such as a difference in means/proportions, the two approaches are functionally equivalent, and so this aspect of the estimand does not need to be specified.

For both marginal and cluster specific summary measures, either individual average weighting or cluster average weighting can be used (or other weightings, such as cluster period average). For example, the illustrations above are based on individual average weighting—that is, the odds ratio of 5.4 above is the marginal, individual average odds ratio. This value broadly corresponds to the odds ratio that would typically be used in individually randomised trials (ie, the odds ratio that is obtained by calculating the odds across all individuals in each treatment condition, and contrasting these odds between the treatments).

A more detailed overview of marginal and cluster specific (as well as individual average and cluster average) estimands is provided by Kahan et al.^23^

The distinction between marginal and cluster specific summary measures only applies for non-collapsible summary measures, such as the odds ratio (box 2).^23^ ^59^ ^60^ ^61^ ^62^ For collapsible summary measures, such as a difference in means or proportions, the distinction does not apply, because it is functionally equivalent to summarise and contrast across clusters as within clusters. Therefore, for collapsible summary measures, this distinction does not need to be defined.^23^

As mentioned above, for both marginal and cluster specific summary measures, either individual average or cluster average weighting can be used (or other weightings, such as cluster period average). For example, a marginal individual average effect would involve summarising potential outcomes across all individuals, irrespective of cluster, with equal weight given to each individual. Conversely, a marginal cluster average effect would involve summarising potential outcomes across all individuals, irrespective of cluster, but each individual’s potential outcome would be weighted according to the size of their cluster. Thus, both aspects of the summary measure need to be defined, where applicable (eg, marginal individual average, marginal cluster average).^23^ A more detailed overview of marginal and cluster specific (as well as individual average and cluster average) estimands is given by Kahan et al.^23^

## Discussion

The CRT-Estimands Framework presents five attributes that should be described when defining estimands for cluster randomised trials. It builds directly on the estimands framework introduced in the ICH E9(R1) addendum, which was developed primarily with individually randomised trials in mind. While the principles and structure set out in ICH E9(R1) remain applicable, their implementation in cluster randomised trials requires additional considerations due to unique features of these trials. The CRT-Estimands Framework applies to any type of cluster randomised trial, including stepped wedge and cluster crossover trials. Investigators conducting cluster randomised trials should now use the CRT-Estimands Framework in place of the ICH E9(R1) attributes.

Adopting this guidance will help interest holders understand the trial’s research question and consequently its estimand, and to determine whether the trial methods are appropriate for the chosen estimand. Through careful specification of the estimand, this guidance can also help trial investigators choose the trial methods (including data collection, trial conduct, and statistical analysis methods) that are aligned to their research question, which can improve the validity of results and help ensure trials are of most use to interest holders.

An estimand should be described for each trial outcome, and for some outcomes more than one estimand may be of interest. Estimands can be described using a table format, similar to table 3, or in a narrative summary, similar to the last row of table 3. The CRT-Estimands Framework can be used to describe estimands in either format. This framework can be used in conjunction with relevant reporting guidelines, such as the CONSORT (Consolidated Standards of Reporting Trials) and SPIRIT (Standard Protocol Items: Recommendations for Interventional Trials) 2025 statements,^63^ ^64^ along with relevant extensions for cluster, stepped wedge, and cluster crossover trials,^17^ ^18^ ^65^ of which the extension for cluster crossover trials recommends reporting of estimands. While CONSORT and SPIRIT 2025 do not mandate the use of estimands, they do encourage the reporting of estimands when used.^12^ ^66^ For instance, CONSORT 2025 states that “If the trial was designed using the estimands framework, the objectives should be defined in terms of this framework.”^64^ The CRT-Estimands framework provides clear guidance on how to appropriately define and report estimands for these trials.

For some trials, it may be useful for investigators to consider additional issues in the estimand definition that are not explicitly listed within the CRT-Estimands Framework. For instance, the effectiveness of some interventions may be expected to vary according to the time of year they are used (eg, summer *v* winter). This issue is not specific to cluster randomised trials, and could occur in individually randomised trials as well. However, in certain cluster randomised trial designs—such as stepped wedge trials, or parallel group cluster randomised trials with staggered recruitment of clusters—the time of year (calendar time) may be associated with intervention assignment, such that the intervention is used more often during certain seasons. Therefore, when calendar time is expected to influence the intervention’s effectiveness, it may be useful in such designs to consider how calendar time is incorporated into the estimand definition.^49^ ^50^ Of note, the effectiveness of some interventions could vary either by calendar time, by the duration of exposure to the intervention (as discussed in box 5), or both. Therefore, for the designs listed above, it may be necessary to consider both aspects in the estimand definition.

The scope of this guidance is limited to setting out a framework for defining estimands in cluster randomised trials to ensure that they are clearly described and unambiguous and thus does not consider how to choose between different estimands, or how to choose a statistical analysis approach for a given estimand. Both of these topics are active research areas, and we anticipate further guidance on these issues will be forthcoming. However, some guidance is currently available for choosing between different aspects of an estimand, such as between individual average or cluster average effects.^31^ There is also guidance for estimating particular types of estimands, including those based on different weighting schemes^23^ ^24^ ^28^ ^30^ ^31^; summary measures, such as marginal versus cluster specific^23^; and approaches for handling different exposure durations.^47^ ^48^ ^49^ Finally, guidance also exists for estimating certain estimands in stepped wedge and cluster crossover trials, as well as for parallel group, cluster randomised trials with a baseline period.^49^ ^58^ ^67^

### Conclusions

The CRT-Estimands Framework extends the ICH E9(R1) addendum to account for the additional needs of cluster randomised trials. Use of the framework should improve the clarity of estimands in cluster randomised trials, just as the ICH E9(R1) addendum has done for individually randomised trials, and ultimately should help interest holders to make better informed decisions based on these trials.

## Data Availability

The datasets used and/or analysed during the Delphi study are available from CLA on reasonable request (catherine.auriemma@pennmedicine.upenn.edu).

## Web extra

Extra material supplied by authors

Web appendix: Supplementary material

## Notes

Contributors: CLA and FL contributed equally. They took shared responsibility for the execution of the study, including planning and developing content for the Delphi survey and consensus meeting, interpretation of data, and critical input into the manuscript. BCK, FB, AC, MOH, GSC, MB, DB, CLA, and FL participated in the concept and design of the work. All the authors participated in the acquisition, analysis, and interpretation of data, and in the critical revision of the manuscript for important intellectual content. All authors read and approved the final manuscript. BCK is the guarantor. The corresponding author attests that all listed authors meet authorship criteria and that no others meeting the criteria have been omitted.

Patient and public involvement: One patient and public involvement partner (DS) was a consensus meeting participant and is a coauthor of this manuscript.

Dissemination to participants and related patient and public communities: The CRT-Estimands Framework will be disseminated through the CRT-Estimands website (crt-estimand.com) where materials will be freely accessible.

Provenance and peer review: Not commissioned; externally peer reviewed.
