# Supplementary material for: CRT-Estimands Framework: consensus based extension of the ICH E9(R1) addendum for cluster randomised trials
Source: BMJ. 2026 May 21;393:e089050. doi: 10.1136/bmj-2025-089050 (PMC13191442; doi:10.1136/bmj-2025-089050)
Supplement: Supplementary file 1 — Web appendix: Supplementary material [file kahb089050.ww.pdf]

Supplementary material for "*CRT-Estimands Framework: consensus based extension of the ICH E9(R1) addendum for cluster randomised trials*"

Section 1: Methods

We developed this guidance by undertaking: (a) a scoping review to identify a comprehensive list of items for consideration for inclusion in the guidance; (b) a three-round Delphi process to obtain feedback on the importance of each item; (c) a consensus meeting to finalise the items to be included in the guidance; and (d) guidance finalisation, to select the wording and format of the guidance.

Scoping review

The objective of the scoping review was to generate a comprehensive list of candidate items (i.e., suggestions for additions to, or modifications of, the existing ICH E9(R1) attributes) for consideration for inclusion in the guidance. Full details are available in reference 29 listed in the main manuscript.

Briefly, we searched relevant databases (EMBASE and MEDLINE via Ovid, Scopus, and Web of Science) for articles which discuss the definition of estimands related to cluster randomised trials. In addition to articles identified through the search, we also included articles from the personal libraries of the authors.

Articles were eligible for inclusion if they discussed an aspect of the estimand definition for cluster randomised trials which was not explicitly included in one of the five attributes listed in ICH E9(R1) (i.e., something which implied that an addition to, or modification of, the existing ICH E9(R1) attributes may be necessary for the appropriate definition of estimands in CRTs).

Delphi study

The objective of the Delphi study was to obtain feedback on the importance of each item and to develop consensus as to which items were essential. Full details are available in reference 36 listed in the main manuscript.

Briefly, each item identified in the scoping review was rated for importance using a 9-point Likert scale (1-3 = not important; 4-6 = important but not critical; 7-9 = critical). In addition, an 'unable to rate' option was provided.

Participants were able to provide free-text comments for each item to explain the rationale behind their scoring. In addition, in the first-round participants were able to suggest additional items for consideration.

There were three rounds in total:

- **First round**: participants were asked to: (a) rate each item; (b) provide free-text feedback explaining their ratings, if desired; and (c) suggest additional items, if desired
- **Second round**: participants were able to: (a) view scores from the previous round and a summary of feedback from other participants explaining their ratings; (b) rate each item from Round 1 again; (c) rate any new items added after the first round, and (d) provide free-text feedback explaining their ratings, if desired

- **Third round**: participants were able to: (a) view scores from the previous round and a summary of feedback from other participants explaining their ratings for new items introduced at Round 2; (b) rate each new item introduced at Round 2 again, and (c) provide free-text feedback explaining their ratings, if desired

Thus, each item was rated twice, with the items identified from the scoping review being rated in Rounds 1 and 2, and additional items suggested in Round 1 being rated in Rounds 2 and 3. Items suggested by participants during the first round were reviewed for relevance and overlap by the Executive Committee prior to being added for the second round.

After the 3rd round was complete, items were categorised as follows based on their final rating:

- Consensus critical: ≥70% participants scoring 7-9, and <15% scoring 1-3
- Consensus not important: ≥70% participants scoring 1-3, and <15% scoring 7-9
- No consensus: everything else

Results from the Delphi study were then used to inform discussions during the consensus meeting; however, results from the Delphi survey were considered non-binding, with final decisions about the guidance being made during the consensus meeting.

We invited participants with a broad range of expertise to participate in the Delphi survey, including those with methodological expertise in cluster randomised trials; those with practical experience undertaking cluster randomised trials; those with expertise in estimands; and those with other experience deemed relevant, for instance in guideline development, journal editors, etc. We employed a snowball sampling approach, where we invited participants to recommend additional participants to recruit.

Consensus meeting

The objective of the consensus meeting was to finalise the items for inclusion in the guidance.

Members of the consensus meeting panel were invited by the Executive Committee. The aim was to include members who had a range of expertise relevant to the topic. This included members with expertise related to estimands, cluster randomised trials, and guidance development, as well as interest holders who would likely interact with the guidance in practice, including journal editors, trial statisticians, clinicians, and Patient and Public Involvement representatives.

The final consensus meeting panel included members with the following expertise:

- <u>Estimands:</u> FB was a member of the ICH E9(R1) writing committee, and BCK, FB, AC, MOH, ABF, PJH, DMM, and FL have published on the topic of estimands
- <u>Methodological issues in CRTs:</u> BCK, AC, MOH, DB, MKC, KRC, ABF, SDH, PJH, KH, JH, DMM, MT, OCU, and FL have published on various methodological issues pertaining to cluster randomised trials
- <u>Estimands in CRTs:</u> BCK, AC, MOH, DB, ABF, SDH, PJH, KH, DMM, MT, and FL have published on the topic of estimands in cluster randomised trials
- <u>Trial statistician for CRTs:</u> BCK, AC, MOH, ABF, PJH, KH, DMM, MT, and FL have all acted as trial statisticians for CRTs

- Clinical experience: KRC, AXG, SDH, JSR, CLA are all medical professionals. KRC, AXG, and SDH have all acted as Principal Investigator on at least one cluster randomised trial.
- Journal editor: MOH is a Deputy Editor for the *American Journal of Respiratory and Critical Care Medicine*, GSC is a Statistical Editor for the *BMJ*, TF is a Research Editor for the *BMJ*, PJH is a Statistical Editor for *JAMA*, JSR is a Deputy Editor for *JAMA*, and MT is a Deputy Editor for *Clinical Trials*.
- Guidance development: BCK led the CONSORT/SPIRIT extensions for factorial trials, FB has been a member of multiple ICH guideline writing committees, GSC is lead of the UK Equator Network, and led the TRIPOD+AI statement, and has been involved in numerous other guidelines, MKC led the CONSORT extension for cluster randomised trials, ABF led the CONSORT extension for cluster-crossover trials, KH led the CONSORT extension for stepped wedge trials, and SH led the CONSORT 2025 statement.
- Patient and Public Involvement: DS has been a PPI representative on numerous projects, including the CONSORT and SPIRIT extensions for surrogate outcomes.

In advance of the meeting, a participant pack was shared. This pack included brief summaries of the project aims, explanations of key topics (including estimands and cluster randomised trials), an explanation of what would occur during the consensus meeting, and a description of each item to be discussed during the meeting as well as the Delphi voting results for that item.

The meeting began with a series of short presentations to: (a) explain the aims of meeting; (b) provide background on estimands and the ICH E9(R1) addendum; (c) describe the need for additional guidance for cluster randomised trials; (d) summarise the pre-meeting work that had been done (including scoping review and Delphi); and (e) describe the process for discussing and voting on items.

When discussing each item, the item was first introduced by the Chair (one of BCK, FL, or AC). The chair explained the rationale for the item, and then summarised the Delphi rating for the item. Finally, meeting attendees discussed the merits of the item. Discussions were moderated by the Chair. At the end of these discussions, meeting attendees (including the Executive Committee) voted on the item's inclusion in the guidance using an anonymous poll, with the consensus threshold set at ≥70% to be included. Attendees included statisticians/methodologists, clinicians, trial investigators, journal editors, guideline developers, and a patient representative.

Guidance finalisation

Following the expert consensus meeting, the Executive Committee held several online meetings to discuss and agree draft formatting and wording of the new or modified attributes from ICH E9(R1). The draft guidance was then circulated to consensus meeting participants for feedback, and was then further revised by the Executive Committee in response to this feedback.

Section 2: Results

Scoping review

Full results of the scoping review are available in reference 29 in the main manuscript. We identified 8 items during the scoping review, and one additional item was added during the Delphi, leading to 9 items being considered in total (eTable 1).

Delphi study

Full results of the Delphi survey are available in reference 36 in the main manuscript. The Delphi survey took place between October 2024 – February 2025 (Round 1 Oct 16th – 31st 2024; Round 2 Nov 20th – Dec 9th 2024; Round 3 Jan 24th – Feb 10th 2025).

73 participants completed the 1st round of the Delphi; 62 completed the 2nd round, and 59 completed the 3rd round.

Overall, the Delphi panel was highly experienced, with around half having over 10 years of experience in clinical trials, and having been involved in 6 or more trials (eTable 2). Most respondents were statisticians (85%), with other roles mentioned including clinical trialist, journal editor, healthcare professional, or health economist. Many respondents reported having expertise in cluster randomised trials (82%), estimands (44%), and guideline development (19%). Respondents were from North America (45%), Europe (36%), and Australia/Oceania (15%).

Final ratings for each item are provided in eTable 1.

Consensus meeting

22 of 23 participants invited to the expert consensus meeting attended. Each of the 9 items was discussed and voted on. A moderated discussion was held for each item, followed by anonymous voting using an electronic poll. Consensus for inclusion was defined a priori as ≥70% agreement.

Results are shown in eTable 3. Four items reached consensus to be included.

Five items did not reach the consensus threshold, and were therefore excluded. Key issues that emerged during discussions about these items were:

- ***Lack of general applicability***: certain items (e.g. "exposure time of clusters and individuals to the intervention", "how interference or spillover effects are handled") were not believed to be general enough for inclusion, as they only apply to a subset of CRTs
- ***Overlap with existing ICH E9(R1) attribute***: certain items (e.g. "exposure time of clusters and individuals to the intervention", "population of individuals under selection or recruitment bias") were believed to be adequately covered by the existing ICH E9(R1) attributes
- ***Insufficient support during Delphi survey***: certain items (e.g. "How clusters that split, merge, or are empty should be handled", "how interference or spillover effects are handled", "population of individuals under selection or recruitment bias") had low support for inclusion during the Delphi survey (given the pre-defined threshold for

inclusion), and thus their inclusion could increase the burden of using the guidance without increasing its value

Guidance finalisation

The four new items were included in the guidance by modifying the existing ICH E9(R1) attributes (i.e. no new attributes were added). The items were included in the guidance as follows:

- One item ("Population of clusters") was merged into the "Population" attribute of ICH E9(R1); and
- Two items ("How individuals and clusters are weighted" and "Whether treatment effects are marginal or cluster-specific") were merged into the "Population-level summary measure" attribute of ICH E9(R1).
- One item ("Strategies for handling cluster-level intercurrent events") was merged into the "Strategies to handle intercurrent events" attribute of ICH E9(R1);

Wording of the new attributes was finalised via discussions between the Executive Committee, and feedback from the consensus meeting participants.

eTable 1 – Delphi ratings for 9 items*

**Item**	**No. rating**	**Mean**	**% rating as not important (1-3)**	**% rating as critical (7-9)**	**Assessment of consensus**
1. How individuals and clusters are weighted in the estimand	62	8.5	0.0	95.2	Essential
2. Population of clusters	62	7.6	0.0	74.2	Essential
3. Population of individuals under selection or recruitment bias	62	6.7	4.8	58.1	No consensus
4. Exposure time of clusters and individuals to the intervention	62	7.2	4.8	74.2	Essential
5. Whether treatment effects are marginal or cluster-specific	62	8.0	0.0	83.9	Essential
6. Strategies for handling cluster-level intercurrent events	62	8.0	0.0	88.7	Essential
7. How interference or spillover effects are handled	61	6.3	6.6	56.5	No consensus
8. How individuals who leave or change clusters should be handled	62	6.8	3.2	62.9	No consensus
9. How clusters that split, merge, or are empty should be handled	59	5.2	4.8	18.6	No consensus

*Denotes the final rating for each item

eTable 2 – Characteristics of Delphi participants. Reproduced from *Kahan, B.C., Bahti, M., Bi, D. et al. Development of a consensus extension of the estimands framework for cluster randomised trials (CRT-estimands): results from an international Delphi study. Trials* ***27****, 70 (2026). https://doi.org/10.1186/s13063-025-09352-1*

Characteristic (n (%))	Participants (N=73)
Job role[a]	
Statistician	62 (85)
Clinical trialist	18 (25)
Journal editor	6 (8)
Healthcare professional	4 (5)
Other	4 (5)
Health economist	1 (1)
Type of expertise[b]	
Cluster randomised trials	60 (82)
Estimands	32 (44)
Guideline development	14 (19)
Other	4 (5)
Prefer not to say	1 (1)
Number of clinical trials involved in	
0	6 (8)
1-2	11 (15)
3-5	17 (23)
6 or more	37 (50)
Missing	2 (3)
Number of years of experience in clinical trials	
No experience	4 (5)
Less than a year	2 (3)
1-5 years	16 (22)
6-10 years	12 (16)
More than 10 years	37 (51)
Missing	2 (3)
Race	
White	55 (75)
Prefer not to say	7 (10)
Asian	6 (8)
Black	3 (4)
Missing	2 (3)
Gender	
Man	38 (52)
Woman	27 (37)
Prefer not to say	6 (8)
Missing	2 (3)
Geographic area of residence	
North America	33 (45)
Europe	26 (36)
Australia/Oceania	11 (15)

**Prefer not to say**	3 (4)

[a] Participants could select more than one option. “Other” included: biostatistician/faculty (1); epidemiologist (1); analytically inclined epidemiologist (1); funder (1). [b] Participants could select more than one option. “Other” included: trials methodology (1); clinical trials, crossover trials (1); power and sample size for multilevel and longitudinal data, especially continuous (1); did not specify (1).

eTable 3 – Results from expert consensus meeting

Item	n/N (%) voting "Yes"	Voted to include?
1. How individuals and clusters are weighted in the estimand	19/21 (91)	YES
2. Population of clusters	20/21 (95)	YES
3. Population of individuals under selection or recruitment bias	1/21 (5)	NO
4. Exposure time of clusters and individuals to the intervention	7/21 (33)	NO
5. Whether treatment effects are marginal or cluster-specific	19/20 (95)	YES
6. Strategies for handling cluster-level intercurrent events	21/21 (100)	YES
7. How interference or spillover effects is handled	3/19 (16)	NO
8. How individuals who leave or change clusters should be handled	7/21 (33)	NO
9. How clusters that split, merge, or are empty should be handled	4/21 (19)	NO
